# Decreased Rhamnose Metabolic Flux Improved Production of Target Proteins and Cell Flocculation in *Pichia pastoris*

**DOI:** 10.3389/fmicb.2018.01771

**Published:** 2018-08-02

**Authors:** Chengliang Yan, Xinxin Xu, Xue Zhang, Yuwei Zhang, Yuhong Zhang, Zhifang Zhang, Wei Zhang, Bo Liu

**Affiliations:** Biotechnology Research Institute, Chinese Academy of Agricultural Sciences, Beijing, China

**Keywords:** *Pichia pastoris*, rhamnose-inducible promoter, promoter exchange, rhamnose metabolic, cell flocculation

## Abstract

Previously, several genes, including *LRA1–LRA4* and *LRAR*, involved in rhamnose utilization pathway, were discovered in *Pichia pastoris* GS115; among them, LRA3 and LRA4 were considered as key rate-determining step enzymes. A *P. pastoris* expression platform based on the strong rhamnose-inducible promoter P*_LRA3_* did not meet the demands of industrial application due to poor production of recombinant proteins. To enhance recombinant protein production of this expression platform, a genetically engineered strain, *P. pastoris* GS115m, with decreased rhamnose metabolic flux was developed from *P. pastoris* GS115 by replacement of the rhamnose-inducible promoter P*_LRA4_* with another much weaker rhamnose-inducible promoter, P*_LRA2_*. Grown in MRH and YPR media using rhamnose as the main carbon source, the engineered strain showed decreased growth rate and maximal biomass compared with the parental strain. More importantly, grown in rhamnose-containing MRH and YPR media, the recombinant engineered strain harboring a β-galactosidase gene *lacB*, whose expression was regulated by rhamnose-inducible P*_LRA3_*, yielded substantial increases, of 2.5- and 1.5-fold, respectively, in target protein production over the parental strain. Additionally, grown in MRH and YPR media, the engineered strain had remarkable cell flocculation and rapid sedimentation with the increasing of cell density, providing an effective and convenient separation of the fermentation supernatant from strain cells. The engineered strain is a promising expression host for industrial production of target proteins due to its advantages over the parental strain as follows: (i) improved production of recombinant proteins, and (ii) remarkable cell flocculation and rapid sedimentation.

## Introduction

A previous study preliminarily investigated several rhamnose-inducible promoters in *Pichia pastoris*. Among them, the transcription activities of two promoters, P*_LRA3_* and P*_LRA4_*, were higher than those of other promoters, including *PAS_chr4_0338* promoter (P*_LRA1_*), *PAS_chr4_0339* promoter (P*_LRA2_*), *PAS_chr4_0340* promoter (P*_LRAR_*) (Liu et al., 2016), which indicated that LRA3 and LRA4 were rate-determining step enzymes related to rhamnose metabolism. Recombinant proteins under the control of the two promoters, especially P*_LRA3_*, could be efficiently produced using rhamnose as the inducer (Liu et al., 2016). Thereby, a rhamnose-inducible *P*. *pastoris* expression platform was established based on P*_LRA3_*. In this platform, rhamnose acted as the inducer for the following: (i) the transcription activation of genes involved in the rhamnose metabolic pathway to produce enzymes to metabolize rhamnose as a carbon source for *P. pastoris* growth, and (ii) the transcription activation of P*_LRA3_* to produce target recombinant proteins. It was also demonstrated that both high rhamnose concentrations and long induction durations were helpful for producing high yields of target proteins. Additionally, from the perspective of industrial safety, using edible rhamnose instead of flammable and hazardous methanol as an inducer made this expression system a promising platform for the production of some valuable recombinant proteins, such as food-grade proteins and biopharmaceutical products (Vogl et al., 2013). However, the industrial application of this system is severely limited due to the relatively low yields of target proteins. It is, therefore, necessary to optimize the system to greatly enhance production of target proteins.

To optimize an expression system, several key factors, such as expression host strains, expression vectors, fermentation conditions, fermentation processes, and so on, need to be considered. Expression host strains play significant roles in the massive production of target products, and many expression host strains have been modified for use in industry (Choi et al., 2003; Demain and Vaishnav, 2009; Lee and Park, 2010). Strain engineering, mostly by means of genetic modification, provides an effective approach to improve production of target products (Idiris et al., 2010; Gottardi et al., 2017; Hossain et al., 2017). Recently, metabolic engineering strategies – for example, promoter exchange – have been widely used to rebalance metabolic flux related to synthesis of target products in rational designs of expression host strains (Tai and Stephanopoulos, 2013; Qiu et al., 2014; Willenbacher et al., 2016; Vidya et al., 2017; Wang et al., 2017; Wen et al., 2017). Strain engineering using a metabolic engineering strategy aims to improve production of secondary metabolites of interest, and heterologous recombinant proteins as terminal products were seldom reported or analyzed.

As mentioned above, LRA4 was one of the enzymes determining the rate of rhamnose metabolism in *P. pastoris*, and down-regulation of *LRA4* expression could decrease the metabolic flux of rhamnose metabolism, leading to changes in the profile of *P. pastoris*. One of the expected profiles would be a decreased rhamnose metabolic rate, which would lead to the following two features: (i) a slower growth rate of the engineered strain grown in medium using rhamnose as the main carbon source; and (ii) a longer duration of high concentrations of residual rhamnose in the medium, leading to improved production of recombinant proteins controlled under the rhamnose-inducible P*_LRA3_*. Thereby, the engineered strain would differ from the parental strain in growth profile and production of the recombinant protein. Based on the above assumption, the genetically engineered strain *P. pastoris* GS115m, with altered rhamnose metabolic flux, was generated from *P. pastoris* GS115 via promoter replacement, substituting the rhamonose-inducible promoter P*_LRA4_* with the much weaker rhamnose-inducible promoter P*_LRA2_*. Grown under specific conditions, the engineered strain significantly differed from the parental strain with regard to several factors, such as growth rate, maximal biomass, and cell flocculation and sedimentation. The engineered strain exhibited an obvious comparative advantage over the parental strain in yielding recombinant proteins.

## Materials and Methods

### Strains and Media

*Escherichia coli* Trans1-T1 (TransGen, Beijing, China) was used for gene cloning; *P. pastoris* GS115 (American Type Culture Collection: 20864) was the parental strain for the engineered strain and was also the host for gene expression. Plasmid pGHLRA3αLacB, strains *P. pastoris* GS115/*LacB* and *P. pastoris* GS115/*gfp*, in which *lacB* and *gfp* expression is regulated under P*_LRA3_*, was described in a previous study (Liu et al., 2016). *P. pastoris* GS115, *P. pastoris* GS115/*LacB*, or *P. pastoris* GS115/*gfp* were used as the control strain for *P. pastoris* GS115m, *P. pastoris* GS115m/*LacB*, or *P. pastoris* GS115m/*gfp*, respectively, in this study. All strains used in this study are listed in **Table 1**.

**Table 1:** Strains used in this study.

Strain	Characteristics	Source
*E. coli* Trans1-T1	Gene cloning host	CD501, TransGen, Beijing, China
*P. pastoris* GS115	Gene expressing host	ATCC 20864
*P. pastoris* GS115/Δ*LRA4*	*P. pastoris* GS115, Δ*LRA4*	Liu et al., 2016
*P. pastoris* GS115/LacB	*LacB* expressed *P. pastoris* GS115	Liu et al., 2016
*P. pastoris* GS115m	*P. pastoris* GS115 with exchange of P*_LRA4_* against P*_LRA2_*	This study
*P. pastoris* GS115m/LacB	*LacB* expressed *P. pastoris* GS115m	This study

MD and MR media contained 1.34% yeast nitrogen base, 4 × 10^−5^% biotin, and 2% dextrose for MD or 2% rhamnose for MR. MRH and MDH media contained all the components in the MD and MR media, respectively, as well as 0.004% histidine. YPR and YPD media contained 1% yeast extract, 2% peptone, and 2% rhamnose for YPR or 2% dextrose for YPD. To prepare solid medium, agar was supplemented into the above media to a final concentration of 2% (w/v).

The primers used for PCR are listed in **Table 2**.

**Table 2:** Primers used in this study.

Primer	Sequences (5′ to 3′)	Primers used for
C0341up-F	GAAAATTGAATCTCGTGAAGAACC	Amplifying a 570-bp DNA fragment of upstream of the ATG for the initiating methionine of *LRA4*
C0341up-R	TTTTGAAGCTATGGTGTGTGGGTGGTTGGAGTAACCGGGAAG	Amplifying a 570-bp DNA fragment of upstream of the ATG for the initiating methionine of *LRA4*
Zeoncin-F	CCCACACACCATAGCTTCAA AA	Amplifying zeocin resistant gene from plasmid pPICzα
Zeoncin-R	AGCTTGCAAATTAAAGCCTTCG	Amplifying zeocin resistant gene from plasmid pPICzα
P*_LRA2_*-F	CGAAGGCTTTAATTTGCAAGCTGTTGTGTAAGAACTGCGTAATCGAC	Amplifying putative P*_LRA2_*
P*_LRA2_*-R	GGAGCAGGAGGTACTGGAGACATGATTTGCAACCACGGACCTT	Amplifying putative P*_LRA2_*
C0341-F	ATGTCTCCAGTACCTCCTGCTCC	Amplifying the whole open reading frame and putative transcription terminator of *LRA4*
C0341-R	GCTTTTATTACATCCAACGGTGA	Amplifying the whole open reading frame and putative transcription terminator of *LRA4*
LRA4-PE-F	CCACAGCAAGTTAATCCCCAG	Verifying promoter exchange of *LRA4*
LRA4-PE-R	CATCCCATTATACCAAACAGAGCA	Verifying promoter exchange of *LRA4*
0339-F	TTCAATCCACCGAGCCACAG	Real-time PCR for *PAS_chr4_0339*
0339-R	CCAGCTCTTTCTGCCCGTATC	Real-time PCR for *PAS_chr4_0339*
*LRA4*-F	CTTGAATCTCCTTGAAGTAGTGCTC	Real-time PCR for *LRA4*
*LRA4*-R	GGCGTCATTGGAATCAAGAAG	Real-time PCR for *LRA4*
*LRA3*-F	TTCAGTGTCATCTGGGTGCAAC	Real-time PCR for *LRA3*
*LRA3*-R	CCTGACTTCCCACTGATGGTAGAC	Real-time PCR for *LRA3*
GAP-F	GTGGTCATCAAACCGGACTCA	Real-time PCR for *gapdh*
GAP-R	CAAGAAGGTCGTCATCACTGCTC	Real-time PCR for *gapdh*

### Construction of Different *LRA4*-Complemented Strains With *LRA4* Disruption

It was previously confirmed that the *LRA4*-disrupted strain could not survive on rhamnose as the sole carbon source. Plasmids harboring different lengths of P*_LRA3_* (210, 140, 120, and 100 bp) were constructed based on the pGHLRA3 plasmid, and *LRA4* was then ligated into these vectors via the *SnaB*I and *Not*I restriction sites. Also, 2 μg DNA of each resultant plasmid digested by *Swa*I were transformed into the *LRA4*-disrupted strain, and positive transformants were screened on MR medium and further verified by PCR.

### Construction of the Engineered Strain *P. pastoris* GS115M With Altered Rhamnose Metabolic Flux

A 220-bp DNA fragment, designated as P*_LRA2_*, comprising putative P*_LRA2_*, and a 1130-bp DNA fragment, designated as C0341, containing the whole open reading frame and putative transcription terminator of *LRA4*, were amplified from genomic DNA of *P. pastoris* GS115 using primer pairs P*_LRA2_*-F and P*_LRA2_*-R and C0341-F and C0341-R, respectively. Then, the two DNA fragments, P*_LRA2_* and C0341, were fused into the expression cassette E0341, in which P*_LRA2_* was located upstream of the start codon of *LRA4*. The zeocin-resistant gene (*zeocin*), a selective marker for screening positive *P. pastoris* transformants, was obtained from the pPICzα plasmid via PCR using primers zeoncin-F and zeoncin-R. C0341up, a 570-bp DNA fragment upstream of the start codon of *LRA4*, was amplified by PCR using C0341up-F and C0341up-R as primers. Finally, the three fragments, C0341up, *zeocin*, and E0341, were ligated into a DNA fragment by overlap-extension PCR. The resultant DNA fragment was introduced into *P. pastoris* GS115 via electroporation. Positive *P. pastoris* GS115m transformants were screened on YPD medium containing zeocin (100 μg/ml) and verified by PCR and DNA sequencing.

### Preparation of a Recombinant Engineered Strain Expressing *LACB* or *GFP*

*Pichia pastoris* GS115m cells were cultured in YPD medium to an OD_600_ of 1.3∼1.5 to prepare electrocompetent cells using the *Pichia* Expression Kit (catalog no. K1710-01). We introduced 5 μg of the pGHLRA3αLacB or pGHLRA3gfp plasmids, linearized by restriction enzyme *Swa* I, into *P. pastoris* GS115m cells by electroporation. The cells were then spread on MD medium and grown at 28°C to form colonies.

To screen the *LacB*-expressing recombinant engineered strain, transformants were carefully picked and transferred to MR medium with 40 μl of 40 g/L X-gal at 28°C for 48 h. Colonies with blue plaques were the β-galactosidase-producing strain *P. pastoris* GS115m/LacB. To screen for the *gfp*-expressing recombinant engineered strain, transformants were grown in YPD medium; chromosomal DNA was then prepared as templates for amplification of *gfp* by PCR. The transformants harboring *gfp* in their chromosomal DNA were considered positive transformants *P. pastoris* GS115m/*gfp*.

### Total RNA Preparation and Real-Time PCR

Three strains, *P. pastoris* GS115, *P. pastoris* GS115/*LacB*, and *P. pastoris* GS115m/*LacB*, were grown in YPR to an OD_600_ of about 12.0. The cells were harvested by centrifugation (12,000 *g* × 5 min) at 4°C and immediately stored at −80°C. Preparation of total RNA from different *P. pastoris* cells, removal of trace DNA, cDNA synthesis, and real-time PCR assays were performed using previously described methods (Liu et al., 2016).

### Analysis of Growth of Different *P. pastoris* Strains

A single colony from each strain (*P. pastoris* GS115, *P. pastoris* GS115m, and *P. pastoris* GS115/Δ*LRA4*) was inoculated into YPD medium and grown for 48 h at 28°C with shaking. The cultures were centrifuged, washed twice with sterile water, suspended in the same volume of sterile water, inoculated into 200 ml of fresh medium (MRH, MDH, YPR, or YPD) at 1% (v/v), and then grown at 28°C with shaking. Every 12 h, the wet cell weight (WCW) of the cultures was measured and recorded to analyze growth rate and cell biomass. WCW was estimated as follows: 10 ml of fermentation cultures was sampled into preweighed 15 ml tube and was centrifuged at 10,000 *g* for 2 min. The supernatants were discarded, and the tube was reweighed. WCW was then calculated.

Residual rhamnose concentration in medium was measured at A_540_ by 3,5-dinitrosalicylic acid (DNS) assay (Miller, 1959).

### *LACB* Expression and Enzyme Activity Assay

A single colony of different *lacB*-harboring *Pichia* cells was inoculated into YPD medium and cultured for 36 h at 28°C with shaking at 200 rpm. Cultures were inoculated into MR or YPR medium at 1% (v/v) and then grown at 28°C with shaking (200 rpm). The activity and production of β-galactosidase in the culture supernatant were determined at intervals.

β-Galactosidase activity was assayed as follows: 800 μl of 0.25% (w/v) ortho-nitrophenyl-β-galactoside (*o*NPG) dissolved in phosphate-citrate buffer (50 mM, pH 5.2) were pre-incubated at 60°C for 5 min; then 200 μl of enzyme solution were added, and incubated at 60°C for another 15 min; 1 ml of 10% trichloroacetic acid was then used to terminate the reaction, followed by the addition of 2 ml of 1 M Na_2_CO_3_. The absorbance of the solution was measured at 420 nm. Under standard conditions (pH 5.2, 60°C, 15 min), the amount of enzyme releasing 1 μmol of *o*-nitrophenol per minute was defined as one unit of β-galactosidase.

### *GFP* Expression and Determination of Green Fluorescence Intensity

Recombinant *Pichia* cells harboring *gfp* were inoculated into YPD medium and cultured overnight at 28°C with shaking at 200 rpm. Cultures were then transferred into BMRY medium at 1% (v/v), followed by incubation for 48 h at 28°C with shaking. Cells were collected by centrifugation at 8,000 *g* for 2 min, washed once with sterile water, and then resuspended in 0.9% NaCl solution. Green fluorescence intensity in recombinant cells was monitored with a laser scanning confocal microscope (Nikon, Japan) at 2 s per image to collect a 1024 × 1024-pixel image.

### Observation of Cell Flocculation and Sedimentation of *P. pastoris* Strains

A single colony of each strain (*P. pastoris* GS115, *P. pastoris* GS115m, *P. pastoris* GS115/LacB, and *P. pastoris* GS115m/LacB) was inoculated into YPD medium and then grown at 28°C with shaking at 200 rpm. Cultures from each strain were inoculated into 200 ml of fresh YPR or YPD at 1% (v/v) and then grown for 36 h at 28°C with shaking. We transferred 6 ml of culture from each strain into a bottle, and cell flocculation and sedimentation were observed when standing for different durations.

## Results

### Growth Characteristics of Strains Complemented With *LRA4* Under Control of P*_LRA3_*

It was confirmed that P*_LRA3_* length is positively correlated with transcription level and production of target proteins when the length is less than 210 bp (Liu et al., 2016). Additionally, the production of LRA4, which is a rate-limiting enzyme in rhamnose metabolism, would be expected to determine the growth rate of *Pichia* cells. To verify the relationship between the growth rate of *Pichia* cells and LRA4 production, the growth rates of *Pichia* cells in which the expression of *LRA4* was under the control of different 5′-deleted P*_LRA3_* were investigated in *LRA4*-complemented strains.

**FIGURE 1:**
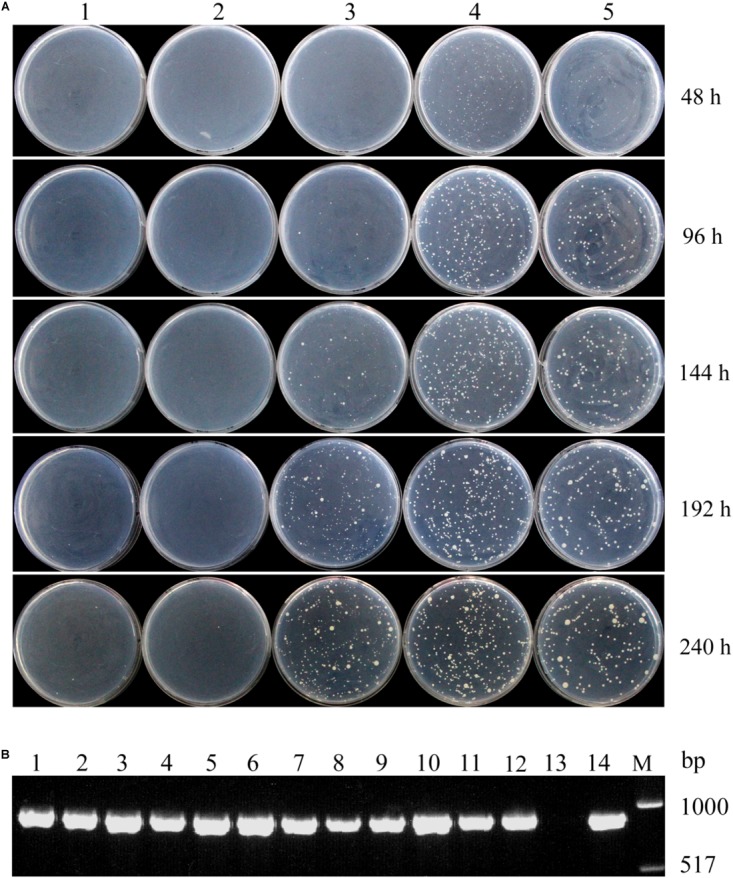
Growth profiles of different strains complemented with *LRA4* under various P*_LRA3_* lengths. **(A)** Colonies formed by *LRA4*-disrupted strains transformed by different plasmids harboring various lengths of P*_LRA3_* controlling the expression of *LRA4*. 1, *LRA4*-disrupted strain; 2, 3, 4, and 5, colonies formed by LRA4-disrupted strains complemented with *LRA4* under control of various lengths of P*_LRA3_*: 100, 120, 140, and 210 bp, respectively. **(B)** Verification of positive transformants via PCR. An 890-bp DNA fragment of *LRA4* was amplified from chromosomal DNA of different strains. Lanes 1–4, 5–8, and 9–12, strains complemented with *LRA4* under control of various lengths of P*_LRA3_* (210, 140, and 120 bp, respectively); lane 13, *LRA4*-disrupted strain; lane 14, wild-type *Pichia pastoris* strain; lane M, DNA marker.

Colonies of transformants harboring long DNA fragments, 140- and 210-bp, containing P*_LRA3_* controlling the high-level expression of *LRA4* were clearly visible on MR medium after a 48-h incubation, and the number of transformants was steady from 48 to 240 h (**Figure 1A**). Meanwhile, transformants harboring a short DNA fragment, 120-bp, containing P*_LRA3_* directing the low expression of *LRA4* grew until 144 h, and the number of transformants increased afterward (**Figure 1A**). In addition, an *LRA4*-disrupted strain and an *LRA4*-disrupted strain containing a 100-bp DNA fragment of P*_LRA3_* to regulate *LRA4* expression did not form colonies during the test (**Figure 1A**). The above results showed that growth rates of *LRA4*-complemented strains were indeed dependent on the length of P*_LRA3_*. As the length of P*_LRA3_* determined its transcription activity, in other words, the growth rates of *LRA4*-complemented strains relied on the transcription activities of P*_LRA3_* with different length. Therefore, exchange of promoters of key genes to change expression levels of key enzymes would to some extent affect physical and physiological profiles of the engineered strains.

To further confirm that these transformants were *LRA4*-complemented strains, chromosomal DNA from 12 transformants harboring different lengths of P*_LRA3_* (210, 140, and 120 bp) were extracted and used as templates for PCR to amplify *LRA4*. Expected PCR products with a molecular weight of about 900 bp were successfully amplified (**Figure 1B**), indicating that these transformants harbored *LRA4*.

### Decreased Expression of *LRA4* in the Engineered Strain With Perturbed Rhamnose Metabolic Flux

To further confirm the relationship between *LRA4* expression level and the growth rate of *Pichia* cells and to investigate the effects of rhamnose metabolic flux rebalance on *P. pastoris*, the engineered strain *P. pastoris* GS115m was developed via a promoter exchange in which *LRA4* expression was controlled by the much weaker (compared with its native promoter P*_LRA4_*) promoter P*_LRA2_*.

**FIGURE 2:**
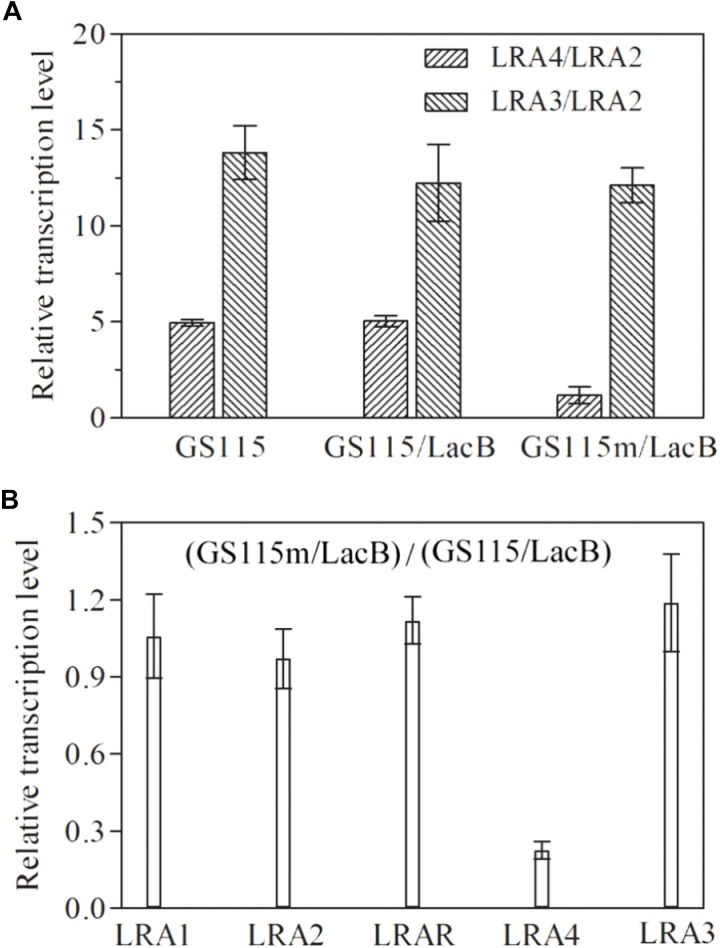
Relative transcription levels of rhamnose utilization related genes (*LRA1*, *LRA2*, *LRAR*, *LRA3*, and *LRA4*) in various strains grown in YPR. **(A)** The ratios of *LRA4*/*LRA2* and *LRA3*/*LRA2* in GS115, GS115/LacB, and GS115m/LacB are shown on the vertical axis. **(B)** Relative transcription levels of *LRA1*, *LRA2*, *LRAR*, *LRA3*, and *LRA4* in GS115m/LacB, using these in GS115/LacB as controls. The GAPDH gene was used as a reference, and the relative expression value of *LRA2* in each strain was designated as 1. Each test was performed in triplicate, and the results are presented as means ± SD of three replicates. GS115, *P. pastoris* GS115; GS115/LacB, *P. pastoris* GS115 harboring *lacB*; GS115m/LacB, *P. pastoris* GS115m harboring *lacB*.

The transcription profiles of genes related to rhamnose metabolism, such as *LRA1*, *LRA2*, *LRA3*, *LRAR*, and *LRA4*, in several related strains were examined using real-time PCR. In *P. pastoris* GS115 and the recombinant strain *P. pastoris* GS115/LacB grown in medium containing rhamnose as the carbon source, *LRA4* transcription levels were about fourfold higher than those of *LRA2*; however, in the engineered *P. pastoris* GS115m/LacB strain, it was almost equal to that of *LRA2*; as a control, *LRA3* in each strain remained steady with higher transcription levels than *LRA2* (**Figure 2A**). Simultaneously, transcription profiles of genes *LRA1*, *LRA2*, *LRA3*, *LRAR*, and *LRA4* in *P. pastoris* GS115/LacB and *P. pastoris* GS115m/LacB were also investigated. The transcription level of *LRA4* in *P. pastoris* GS115m/LacB greatly decreased while transcription profiles of other genes keep similar in the two strains (**Figure 2B**).

These results suggested that the expression of *LRA4* in the engineered strain was greatly down-regulated after promoter exchange, and the engineered strain developed as expected.

### Growth Profiles of the Engineered Strain

In the rhamnose-inducible promoter-based *P. pastoris* expression platform, rhamnose plays two roles: it served as a carbon source for *P. pastoris* growth and acted as an inducer for the transcription activation of P*_LRA3_* to produce recombinant proteins. It was proposed that rhamnose metabolism affected these two processes. Due to down-regulation of *LRA4* expression, we expected the rhamnose metabolic rate of *P. pastoris* GS115m to decrease compared with that of the parental strain *P. pastoris* GS115, leading to the slower growth of *P. pastoris* GS115m when rhamnose was used as the sole carbon source.

**FIGURE 3:**
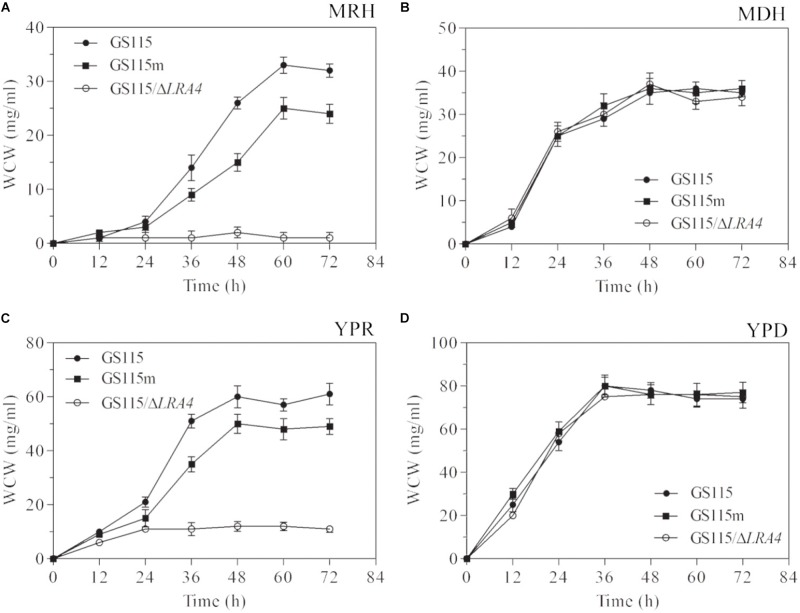
Growth characteristics of strains when grown in media MRH **(A)**, MDH **(B)**, YPR **(C)**, or YPD **(D)**. WCW, wet cell weight. GS115, *P. pastoris* GS115; GS115m, *P. pastoris* GS115m; GS115/Δ*LRA4*, *P. pastoris* GS115 with *LRA4* disruption. Each test was performed in triplicate, and the results are presented as means ± SD of three replicates.

To verify the above hypothesis, the growth profiles of three strains, *P. pastoris* GS115, *P. pastoris* GS115m, and *P. pastoris* GS115/Δ*LRA4*, which cannot utilize rhamnose as a carbon source, grown in different media were analyzed. The specific growth rate of *P. pastoris* GS115m grown in MRH medium, which contained 2% (w/v) rhamnose as the sole carbon source, was low compared with that of the parental strain, 0.07 vs. 0.124 h^−1^, although the two strains could survive on L-rhamnose (**Figure 3A**). It was simultaneously observed that the maximal biomass (25 mg/ml) of *P. pastoris* GS115m was much lower than that of the parental strain (33 mg/ml), and the maximal biomass of *P. pastoris* GS115m was 75.8% of that of the parental strain (**Figure 3A**). Grown in YPR medium using rhamnose as the main carbon source, the specific growth rates of *P. pastoris* GS115m and the parental strain were also different, 0.131 vs. 0.149 h^−1^, and the maximal biomass of *P. pastoris* GS115m was 80.3% of that of the parental strain (**Figure 3C**). However, no obvious differences in the growth rate and maximal biomass of the two strains were observed when they were grown in the following media: MDH using glucose as the sole carbon source and YPD using glucose as the main carbon source (**Figures 3B,D**). Based on these results, when rhamnose was the main carbon source, the perturbation in rhamnose metabolic flux affected *P. pastoris*; its effects included, but were not limited to, the growth rate and the maximal cell biomass.

**FIGURE 4:**
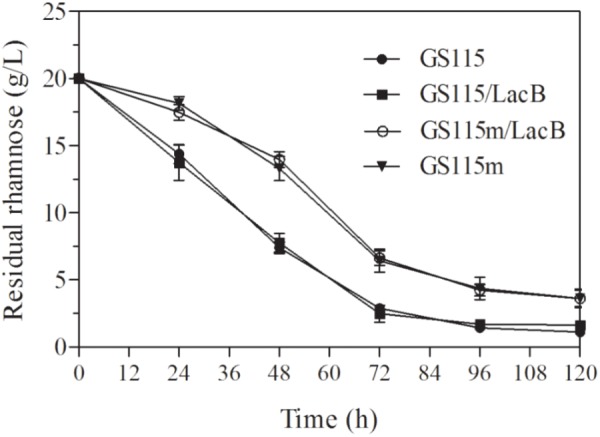
Concentrations of residual rhamnose in cultures of strains grown in YPR at different duration. GS115, *P. pastoris* GS115; GS115/LacB, *P. pastoris* GS115/LacB; GS115m, *P. pastoris* GS115m; GS115m/LacB, *P. pastoris* GS115m/LacB. Each test was performed in triplicate, and the results are presented as means ± SD of three replicates.

The concentrations of residual rhamnose in the cultures of the engineered strains presented higher than these in two non-genetically engineered strains, *P. pastoris* GS115 and *P. pastoris* GS115/LacB (**Figure 4**), which suggested that down-regulated expression of *LRA4* indeed decreased rhamnose metabolism rate in the engineered strains.

### Enhanced Production of Target Protein in the Engineered Strain

**FIGURE 5:**
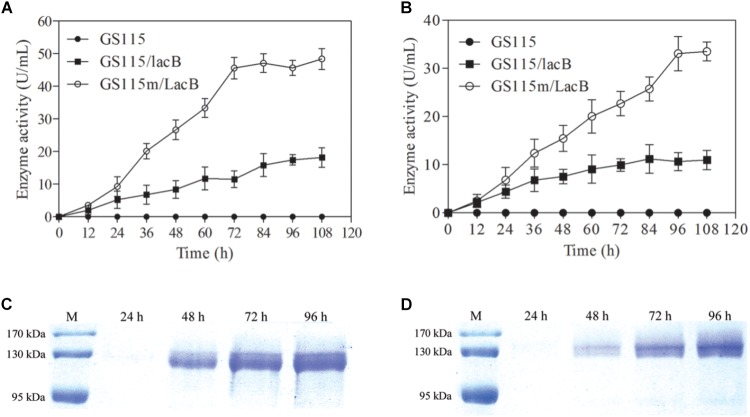
β-Galactosidase production in culture supernatants of different strains grown in different media. β-Galactosidase activity in culture supernatants of strains grown in YPR **(A)** or MRH **(B)**. β-Galactosidase production in culture supernatants of GS115m/LacB **(C)** and GS115/LacB **(D)** in YPR. GS115, *P. pastoris* GS115; GS115/LacB, *P. pastoris* GS115/LacB; GS115m/LacB, *P. pastoris* GS115m/LacB. Each test was performed in triplicate, and the results are presented as means ± SD of three replicates.

The engineered strain differed from the parental strain with regard to growth rate and maximal cell biomass. However, we focused on whether the engineered strain could enhance the production of target proteins. Two *lacB*-harboring strains, *P. pastoris* GS115/LacB and *P. pastoris* GS115m/LacB, were used to examine this feature. The two strains plus *P. pastoris* GS115 were grown in 200 ml of YPR and MRH media, respectively, and the β-galactosidase activities in the supernatant were determined. The β-galactosidase activities in the supernatants from *P. pastoris* GS115/LacB and *P. pastoris* GS115m/LacB cultures increased with incubation time from 0 to 72 h, reaching a maximum at 96 h. The maximal β-galactosidase activities of *P. pastoris* GS115m/LacB in YPR and MRH media yielded 1.5- and 2.5-fold improvements over those of *P. pastoris* GS115/LacB, respectively (**Figures 5A,B**). Estimated according to specific activity of β-galactosidase (575 U/mg), β-galactosidase amounts in the supernatants reached to 84.1 μg/ml for *P. pastoris* GS115m/LacB and 31.6 μg/ml for *P. pastoris* GS115/LacB when grown in YPR medium, and 58.3 μg/ml for *P. pastoris* GS115m/LacB and 19.1 μg/ml for *P. pastoris* GS115/LacB when grown in MRH medium.

Twenty microliter of culture supernatants from *P. pastoris* GS115/LacB and *P. pastoris* GS115m/LacB grown in YPR were loaded for SDS-PAGE, and β-galactosidase amounts were further examined using SDS-PAGE (**Figures 5C,D**). The results were consistent with the β-galactosidase activity analysis. *P. pastoris* GS115m/LacB had a higher yield of recombinant proteins compared to *P. pastoris* GS115/LacB.

**FIGURE 6:**
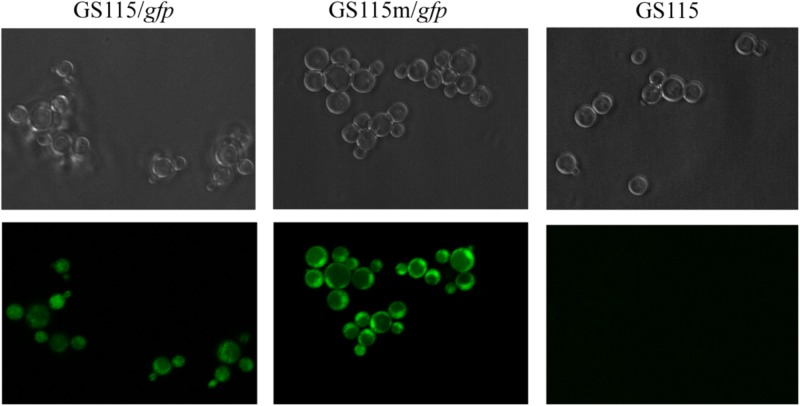
Green fluorescence in different strains grown in YPR. GS115/*gfp*, *P. pastoris* GS115 harboring *gfp*; GS115m/*gfp*, *P. pastoris* GS115 harboring *gfp*; GS115, *P. pastoris* GS115.

To further investigate the expression of recombinant proteins in the engineered strain, the intracellular expression of *gfp* was monitored in different strains grown in YPR (**Figure 6**). Mean green fluorescence intensity in *P. pastoris* GS115/gfp and *P. pastoris* GS115m/gfp was 573 and 1223, respectively. The results also indicated that the expression of the target gene in the engineered strain was higher than that in the wild strain.

### Cell Flocculation and Sedimentation in the Engineered Strains *P. pastoris* GS115M and *P. pastoris* GS115M/LACB

Rhamnose metabolism was important for the survival of *Pichia* cells when used as the sole source of carbon and energy. Changes in the rhamnose metabolic flux in *Pichia* cells may lead to numerous physiological alterations in addition to those affecting the growth rate and production of recombinant proteins.

**FIGURE 7:**
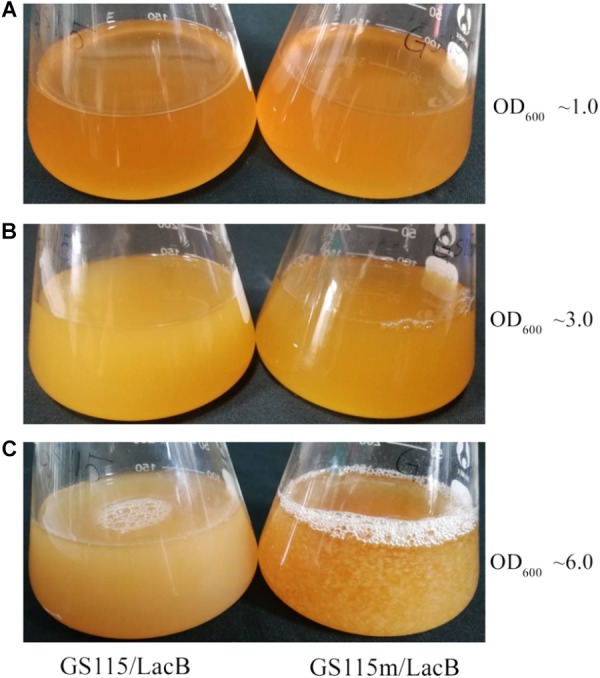
Cell flocculation profiles of GS115/LacB and GS115m/LacB grown in YPR at different cell density. **(A)** OD_600_ ∼1.0; **(B)** OD_600_ ∼3.0; **(C)** OD_600_ ∼6.0. GS115/LacB, *P. pastoris* GS115/LacB; GS115m/LacB, *P. pastoris* GS115m/LacB.

*Pichia pastoris* GS115/LacB and *P. pastoris* GS115m/LacB were grown in rhamnose-containing medium YPR. The two strains did not present cell flocculation at low cell density (OD_600_ below 3.0) (**Figures 7A,B**); at high cell density (OD_600_ beyond 6.0), the engineered strain presented cell flocculation whereas *P. pastoris* GS115 did not (**Figure 7C**).

**FIGURE 8:**
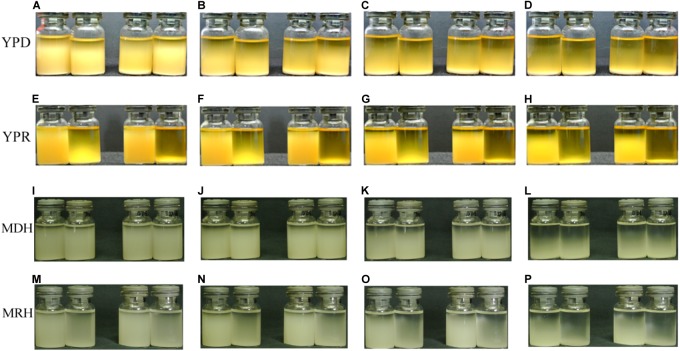
Cell flocculation and sedimentation of different strains in different media. **(A,E,I,M)** 30 min; **(B,F,J,N)** 60 min; **(C,G,K,O)** 120 min; **(D,H,L,P)** 240 min. Cultures from left to right in **(A–P)** are *P. pastoris* GS115, *P. pastoris* GS115m, *P. pastoris* GS115/LacB, and *P. pastoris* GS115m/LacB, respectively.

Cell flocculation accelerated the sedimentation of strain cells. Four strains, *P. pastoris* GS115, *P. pastoris* GS115m, *P. pastoris* GS115/LacB, and *P. pastoris* GS115m/LacB, were grown in YPD or YPR medium for about 36 h at 28°C; then, 6 ml of each culture were transferred into a bottle for flocculation and sedimentation analysis. In YPD medium, the cell flocculation and sedimentation of each strain were similar, and sedimentation was present during all test durations: 30 min (**Figure 8A**), 60 min (**Figure 8B**), 120 min (**Figure 8C**), and 240 min (**Figure 8D**). When cultured in YPR medium, cell flocculation occurred in the engineered strains (*P. pastoris* GS115m and *P. pastoris* GS115m/LacB), and cell sedimentation rates improved. The engineered strains almost fully settled within 30 min (**Figure 8E**), whereas the sedimentation of the parental strains (*P. pastoris* GS115, *P. pastoris* GS115/LacB) was present throughout the test at durations of 30 min (**Figure 8E**), 60 min (**Figure 8F**), 120 min (**Figure 8G**), and 240 min (**Figure 8H**). Rapid cell flocculation and sedimentation also happened to the engineered strains (*P. pastoris* GS115m and *P. pastoris* GS115m/LacB) when grown in MRH medium (**Figures 8I–P**), although rates of cell flocculation and sedimentation were low compared these when grown in YPR medium. These results suggested that more pronounced cell flocculation and more rapid sedimentation occurred in engineered strains grown in rhamnose-containing media (MRH and YPR) than in those grown in non-rhamnose containing media (MDH and YPD).

## Discussion

Great progress in metabolic engineering facilitated the engineering of microbes to improve production of target products of interest (Kruyer and Peralta-Yahya, 2017). Most optimize biochemical pathways to redirect metabolic flux; for example, increasing the production of bottleneck enzymes or/and decreasing the byproduct-related gene expression yields high-level production of desired secondary metabolites, including pharmaceuticals, biofuels, and material precursors (Zhang, 2015). This study sought to improve the production of recombinant proteins instead of secondary metabolites via metabolic engineering, and the results provide insight into the engineering of other strains designed to improve the production of recombinant proteins.

To enhance the production of recombinant proteins in a rhamnose-inducible promoter P*_LRA3_*-based *P. pastoris* expression platform, the engineered strain with down-regulated expression of one key rhamnose metabolism-related gene, *LRA4*, was acquired. Decreases in both the growth rate and the maximal cell biomass of the engineered strain occurred in medium MRH using rhamnose as the sole carbon source, which was consistent with the fact that LRA4 is a step-limiting enzyme involved in rhamnose metabolism. Moreover, down-regulated expression of *LRA4* led to slower growth of the engineered strain due to a lower rate of consumption of rhamnose. However, the increases in cell flocculation and in the sedimentation rate of the engineered strains grown in rhamnose-containing media (MRH and YPR) were unexpected. One possible explanation for these findings may be as follows. Cell flocculation, a mechanism for responding to environmental changes, is usually correlated with environmental stress, including limited carbon source availability (Tanneberger et al., 2007). Decreases in the rhamnose metabolic rate, which led to a lower energy supply for cell growth, may have delivered an inaccurate message to the engineered strain to the effect that the carbon source available in the medium was poor, leading cells to flocculate despite the enough availability of rhamnose in the growth medium. Cell flocculation presents numerous advantages in industrial fermentation processes, e.g., providing an effective and convenient separation of the fermentation supernatant from strain cells; and these were comprehensively described by Soares (Soares, 2011). To present, at least four aspects of the engineered strain (the decrease in growth rate, low maximal biomass, remarkable cell flocculation, and rapid sedimentation) resulted from a rebalance of the rhamnose metabolic flux, although the mechanisms underpinning the cell flocculation in rhamnose-containing media, especially YPR, are unknown.

As mentioned in Section “Introduction,” in a rhamnose-inducible expression system, rhamnose acts not only as the carbon source for growth but also as the inducer of the transcription activation of P*_LRA3_* to direct the expression of the gene of interest. It was shown that recombinant protein production closely depends on rhamnose concentration and induction duration, with high rhamnose concentrations and long induction durations helpful for producing high-level yields of target proteins in this expression platform. In the engineered strain, a lower rhamnose metabolic rate resulted in a higher concentration of residual rhamnose. When combined with longer induction duration, this would lead the rhamnose-inducible promoter P*_LRA3_* to produce higher levels of recombinant proteins. This explained the fact that recombinant protein yields by the engineered strain were higher than those in the parental strain.

Previously, it was verified that β-galactosidase expression level under P*_LRA3_* was 40% of that under the most widely used promoter P*_AOX1_* in *P. pastoris* (Liu et al., 2016). In this study, the β-galactosidase production of *P. pastoris* GS115m/LacB in YPR and MRH media presented 2.5- and 3.5-fold of those of *P. pastoris* GS115/LacB, respectively. In other words, production of target proteins under P*_LRA3_* in the engineered strain was comparable with that under P*_AOX1_* in wild strain, and recombinant gene expression level in this expression system had potential for industry.

Rhamnose is much expensive compared with dextrose. It is no doubt that recombinant protein production is cost-competitive if dextrose can be used as the carbon source. To present, dextrose is usually combined with constitutive promoters to produce recombinant proteins in *P. pastoris*. However, some recombinant proteins, which are lethal or toxic to *P. pastoris* growth, cannot be expressed using dextrose combined with constitutive promoters. When lethal or toxic recombinant proteins would be produced, dextrose can be first used as carbon source for *P. pastoris* growth, and then rhamnose is used as the inducer and carbon source to produce recombinant proteins once cell biomass is up to a certain value. So, dextrose and rhamnose are cooperative for producing lethal or toxic recombinant proteins in *P. pastoris*. Additionally, the strict and strong methanol-inducible promoter P*_AOX1_* was usually used for heterologous protein production in *P. pastoris*. However, P*_AOX1_* is not proper for recombinant protein production because of disadvantages of the inducer, methanol: highly flammable and hazardous to health. Using edible rhamnose as the inducer made rhamnose-inducible promoters be excellent in food-grade and therapeutically important recombinant proteins in *P. pastoris*.

In summary, down-regulated expression of *LRA4* encoding a step-limiting enzyme resulted in a decrease in the rhamnose metabolic rate in the engineered strain, leading to several advantageous features for industrial applications, including remarkable cell floccution, rapid sedimentaion, and the high-level productivity of recombinant proteins. Besides, based on a transcriptional activity analysis of P*_LRA3_* and P*_LRA4_*, LRA3 was a more important step-limiting enzyme than LRA4. Strain engineering based on *LRA3*, or/and *LRA4*, would be performed in a future study.

## Author Contributions

BL and WZ designed the study. XZ, CY, XX, and YwZ performed the experiments. YZ, XX, and ZZ analyzed the data. XZ, CY, and XX wrote the manuscript. All authors participated in the discussion of the research and approved the final manuscript.

## Conflict of Interest Statement

The authors declare that the research was conducted in the absence of any commercial or financial relationships that could be construed as a potential conflict of interest.
